# Differential diagnosis of non-hydrocephalus ventricular dilation and hydrocephalus

**DOI:** 10.3389/fneur.2026.1834975

**Published:** 2026-06-17

**Authors:** Ming Li, Guoyi Gao

**Affiliations:** 1 Department of Neurosurgery, Beijing Tiantan Hospital, Capital Medical University, Beijing, China; 2 Beijing Key Laboratory of Central Nervous System Injury, Beijing Neurosurgical Institute, Capital Medical University, Beijing, China

**Keywords:** cerebrospinal fluid biomarkers, cerebrospinal fluid dynamics, clinical symptoms, differential diagnosis, hydrocephalus, neuroimaging, ventricular dilation

## Abstract

Ventriculomegaly is a common structural neuroimaging finding, yet its differentiation from hydrocephalus remains a substantial clinical challenge. Misclassification of non-hydrocephalic ventriculomegaly as hydrocephalus, or of hydrocephalus as simple ventriculomegaly, may result in inappropriate management, including unnecessary shunt surgery or delayed treatment. Accurate discrimination between these entities is therefore critical for optimal clinical decision-making. Based on a review of the major biomedical databases, we summarized current approaches to differentiating non-hydrocephalic ventriculomegaly from hydrocephalus using neuroimaging, cerebrospinal fluid (CSF) dynamics, biomarkers, and clinical symptoms, with the goal of establishing an integrated diagnostic framework. Neuroimaging markers, including the Evans Index (EI), callosal angle (CA), and disproportionately enlarged subarachnoid-space hydrocephalus (DESH), are useful for characterizing the pattern of ventricular enlargement, while advanced modalities such as phase-contrast MRI and diffusion tensor imaging (DTI) further enhance diagnostic performance. Assessment of CSF dynamics through intracranial pressure (ICP) monitoring, lumbar infusion testing, and resistance to CSF outflow (Rout) provides important insights into CSF circulatory dysfunction. In addition, CSF biomarkers such as tau and amyloid-β may assist in distinguishing hydrocephalus from neurodegenerative disease and age-related ventriculomegaly. Although gait disturbance, cognitive impairment, and urinary incontinence represent the classic clinical triad of hydrocephalus, symptom overlap across disorders limits their diagnostic specificity. Clinical assessment should therefore incorporate symptom patterns, temporal evolution, and treatment responsiveness. Overall, accurate differentiation between non-hydrocephalic ventriculomegaly and hydrocephalus requires a multidimensional strategy integrating neuroimaging, CSF dynamics, biomarkers, and clinical symptoms. This comprehensive framework may improve diagnostic accuracy and guide clinical management.

## 1 Introduction

Differentiating non-hydrocephalic ventriculomegaly from hydrocephalus is a clinically significant challenge, particularly in the evaluation and treatment of patients with suspected idiopathic normal pressure hydrocephalus (iNPH), for whom accurate distinction between these entities is essential for appropriate clinical decision-making. INPH is regarded as one of the most treatable causes of cognitive impairment in the elderly (1). Non-hydrocephalic ventriculomegaly represents a structural neuroimaging finding characterized by ventricular enlargement without significant alteration in cerebrospinal fluid (CSF) dynamics, such as passive ventricular expansion secondary to brain parenchymal loss (2). By contrast, hydrocephalus is a pathological condition caused by disturbances in CSF production, circulation, or absorption, leading to pathological ventricular enlargement (3). Failure to distinguish between these entities may lead to inappropriate treatment or delayed intervention, particularly by exposing patients with non-hydrocephalic ventriculomegaly to unnecessary shunt surgery or by delaying timely treatment in patients with hydrocephalus, both of which may substantially compromise quality of life.

Currently, the differential diagnosis between non-hydrocephalic ventriculomegaly and hydrocephalus relies mainly on neuroimaging, CSF dynamics, CSF biomarkers, and clinical symptoms, yet no integrated diagnostic gold standard has been established in clinical practice. Neuroimaging markers, including the Evans Index (EI), callosal angle (CA), and disproportionately enlarged subarachnoid-space hydrocephalus (DESH), together with advanced modalities such as phase-contrast MRI and diffusion tensor imaging (DTI), provide important information for identifying ventricular enlargement and differentiating hydrocephalus from non-hydrocephalic ventriculomegaly (4–8). However, imaging findings alone are often insufficient for a definitive diagnosis. Similarly, CSF dynamic assessments, such as intracranial pressure (ICP) monitoring, lumbar infusion testing, and resistance to CSF outflow (Rout), can distinguish these conditions from a pathophysiological standpoint, but their clinical use remains constrained by limited availability and technical requirements (9). CSF biomarkers, including amyloid-β and tau, have shown considerable potential for detecting coexisting neurodegenerative pathology, particularly in differentiating iNPH from neurodegeneration-related ventriculomegaly, although their widespread clinical application still requires further validation (10). Clinical symptoms are also essential in refining the differential diagnosis. Gait disturbance, cognitive impairment, and urinary incontinence represent the classic triad of hydrocephalus (11). However, symptom overlap may also occur in non-hydrocephalic ventriculomegaly. The response of patients to the CSF tap test may provide additional diagnostic value and help guide clinical management (12).

Despite progress in multidimensional diagnostic approaches, the differential diagnosis between hydrocephalus and non-hydrocephalic ventriculomegaly still lacks a comprehensive framework that integrates all relevant dimensions. This review therefore aims to summarize current diagnostic methods involving neuroimaging, CSF dynamics, CSF biomarkers, and clinical symptoms, and to discuss the strengths and limitations of each modality. We further propose a multidimensional diagnostic pathway for distinguishing ventriculomegaly from hydrocephalus, with the goal of providing a practical reference for clinicians managing patients with suspected hydrocephalus and, ultimately, improving clinical outcomes.

## 2 Methods: literature search strategy and study selection

This article is a narrative review. A comprehensive literature search was conducted in PubMed, Web of Science, and Embase for studies published up to March 2026. Search terms included “ventriculomegaly,” “hydrocephalus,” “diagnosis,” “neuroimaging,” “cerebrospinal fluid dynamics,” “biomarkers,” “gait disturbance,” “cognitive impairment,” and “urinary incontinence.” Studies were included if they: (1) reported clinical data, systematic reviews, or meta-analyses relevant to differentiating non-hydrocephalic ventriculomegaly from hydrocephalus; and (2) provided clearly defined diagnostic criteria or measurable outcomes. Exclusion criteria comprised case reports, non-peer-reviewed articles, studies lacking explicit diagnostic definitions or outcome measures, and those focused on pediatric populations or unrelated pathologies.

Although this review is narrative rather than a formal systematic review, a structured selection process was applied to enhance methodological transparency and reproducibility. This approach allows a comprehensive and reliable synthesis of representative studies while acknowledging the inherent limitations of narrative review methodology. The main purpose of this review is to integrate representative diagnostic methods and qualitatively summarize existing evidence.

## 3 Result

### 3.1 Neuroimaging

Neuroimaging plays a central role in distinguishing non-hydrocephalic ventriculomegaly from hydrocephalus. One of the most widely used indices for assessing ventricular enlargement is the EI, which was first introduced by Evans (4). The EI is calculated as the ratio of the maximal width of the frontal horns to the maximal internal diameter of the skull. In general, an EI ≥ 0.30 is considered indicative of ventriculomegaly (11). However, this threshold alone is insufficient to reliably distinguish hydrocephalus from ventriculomegaly secondary to cerebral atrophy. In a study by Brix et al. (13), substantial variability in EI measurements was observed among healthy older adults, suggesting that the conventional cutoff of 0.30 may not adequately separate normal from enlarged ventricles. The authors proposed a higher threshold of 0.32–0.37 for defining ventriculomegaly, particularly in the elderly. Therefore, EI should always be interpreted in conjunction with other clinical and imaging features. The CA is another widely used imaging marker and is measured on a coronal image at the level of the posterior commissure. Ishii et al. (5) reported that a CA < 90° is useful for differentiating idiopathic normal pressure hydrocephalus (iNPH) from AD-related ventriculomegaly, as patients with iNPH typically show a smaller CA than those with AD or age-related cerebral atrophy. In addition, Cagnin et al. (14) proposed the simplified CA, measured on a coronal slice through the midpoint of the corpus callosum, with the angle formed by the inferior point of the corpus callosum and the lateral borders tangent to the lateral ventricles. A simplified CA < 123° has likewise been shown to aid in distinguishing iNPH from dementia with ventriculomegaly. Nevertheless, CA should not be considered a stand-alone diagnostic marker, as it may be affected by individual anatomical variation and should be interpreted together with other clinical findings. DESH is a characteristic imaging marker associated with idiopathic normal pressure hydrocephalus (iNPH) and has emerged in recent years as a key diagnostic feature (11). It is characterized by a disproportionate enlargement of the subarachnoid spaces, particularly at the Sylvian fissures and high convexity, accompanied by narrowing of the high-convexity sulci and ventricular enlargement. Deng and colleagues reported that the DESH score has important diagnostic value in differentiating idiopathic normal pressure hydrocephalus (iNPH) from ventriculomegaly associated with AD, with a specificity of up to 93% (15). Notably, several studies have reported that the presence of DESH is closely associated with a more favorable response to shunt surgery (16, 17). However, the existing DESH scores still need further standardization and validation through large-scale prospective clinical trials. Recently, composite imaging scales based on multiple radiological markers have also been developed. In the diagnostic model proposed by Fällmar et al. (18) for distinguishing iNPH from a range of neurodegenerative disorders, including VD, progressive supranuclear palsy (PSP), multiple system atrophy parkinsonian type (MSA-P) and healthy controls the callosal angle, enlarged Sylvian fissures, and focally enlarged sulci were found to play major roles in the differential diagnosis. Although the callosal angle showed the highest diagnostic accuracy as a single imaging marker for differentiating iNPH from its mimics, the authors nevertheless recommended the use of a simplified composite imaging scale. In addition, some studies have shown that relatively mild hippocampal atrophy and less prominent para hippocampal fissure dilatation may also help distinguish iNPH from AD (19, 20). Other potentially useful markers include periventricular white matter lesion volume (PWML-V) and deep white matter lesion volume (DWML-V), with DWML-V predominating in iNPH, whereas PWML-V is more commonly observed in Alzheimer’s disease and in healthy individuals (21). Similarly, these methods all require further validation.

Beyond conventional structural imaging, advanced MRI techniques, including phase-contrast MRI and DTI, provide additional information on CSF dynamics and white matter integrity in patients with suspected hydrocephalus. Haggard et al. reported that cerebrospinal fluid peak diastolic velocity (PDV), peak systolic velocity (PSV), and stroke volume (SV) were significantly higher in patients with normal pressure hydrocephalus (NPH) than in those with cerebral atrophy. Specifically, PDV, PSV, and SV were 4.2 cm/s, 4.96 cm/s, and 83.23 μL/cycle, respectively, in the NPH group, versus 1.33 cm/s, 1.66 cm/s, and 11.63 μL/cycle, respectively, in the cerebral atrophy group (22). More recently, a meta-analysis demonstrated that mean flow (MF) remains consistently higher in patients with idiopathic normal pressure hydrocephalus (iNPH) than in control groups, including patients with brain atrophy, with an effect size of 0.143 mL/s (95% CI, 0.131–0.155 mL/s) (23). This imaging modality provides a more dynamic assessment of CSF circulation and may be particularly valuable for identifying hydrocephalus that is not readily apparent on static imaging. DTI can reveal microstructural white matter alterations characteristic of iNPH, including abnormalities in the corpus callosum and internal capsule. These changes are thought to reflect tissue injury related to chronic ventricular enlargement and fluctuations in CSF pressure (24). Kim et al. (8) reported that, on preoperative DTI, patients with iNPH had significantly higher fractional anisotropy (FA) in the posterior limb of the internal capsule than patients with AD, vascular dementia (VD), and healthy controls. Using a cutoff value of 0.613, FA in the posterior limb of the internal capsule yielded a sensitivity of 87.5% and a specificity of 95.0% for distinguishing iNPH. Another study focusing on the corpus callosum suggested that this region may contribute to the differential diagnosis among iNPH, AD, and progressive supranuclear palsy (PSP). Based on the spatial distribution of the principal diffusion direction (V1) derived from DTI, the V1 distribution in the splenium of the corpus callosum differed between iNPH and both AD and PSP, and V1 was more accurate than splenial volume for differential diagnosis (25). In contrast, another study found that patients with iNPH had lower FA values than both the AD group and healthy controls in the bilateral anterior corona radiata, corpus callosum, bilateral superior longitudinal fasciculus, bilateral posterior thalamic radiation, bilateral external capsule, and middle cerebellar peduncles. Patients with iNPH also showed higher mean diffusivity (MD) in the bilateral anterior corona radiata, corpus callosum, bilateral superior longitudinal fasciculus, left posterior thalamic radiation, bilateral external capsule, and middle cerebellar peduncles (26). These parameters may therefore serve as potential imaging markers for distinguishing iNPH from AD. Although these advanced techniques have not yet been routinely adopted in clinical practice, they hold considerable promise for improving the sensitivity and specificity of hydrocephalus diagnosis, particularly in cases in which conventional imaging findings are inconclusive.

In conclusion, both basic brain structural imaging and advanced imaging techniques require prospective, multi-center validation of imaging thresholds and the establishment of standardized scoring systems. Future research should focus on automated image analysis, integration of structural and functional data, and prospective studies that link imaging features with clinical outcomes and shunt responsiveness.

### 3.2 Cerebrospinal fluid dynamics

Altered CSF dynamics is one of the hallmarks of hydrocephalus. In the differential diagnosis of ventriculomegaly, ICP monitoring provides functional information on CSF dynamics beyond that afforded by static neuroimaging. However, static ICP measurements alone are insufficient for the diagnosis of hydrocephalus, as they cannot capture the full complexity of CSF circulation. In contrast, assessment of CSF compliance and pulsatility, particularly through techniques such as lumbar infusion testing, provides more direct evidence of the underlying pathophysiological mechanisms. A study by Tans showed that CT combined with lumbar infusion testing achieved an accuracy of 71% in differentiating normal pressure hydrocephalus (NPH) from cerebral atrophy (27). In another study, Weerakkody et al. (28) performed a systematic analysis of 1,423 patients and found that ICP monitoring mainly distinguishes these conditions through three modalities. First, overnight monitoring can capture the status of compensatory reserve under resting conditions; patients with shunt-responsive NPH often exhibit normal baseline pressure but a persistently elevated RAP index, defined as the correlation coefficient between mean ICP wave amplitude and mean ICP, RAP index > 0.6 accompanied by significant B-wave activity, whereas patients with cerebral atrophy typically show a persistently low RAP index < 0.6 and no significant B waves. Second, infusion testing directly quantifies resistance to CSF outflow (Rout) by infusing artificial CSF into the CSF space at a constant rate. Patients with NPH typically show Rout > 13 mmHg/mL/min, with RAP rising above 0.6 during infusion, indicating impaired CSF absorption and rapid exhaustion of compensatory reserve, whereas patients with cerebral atrophy typically show Rout < 10 mmHg/mL/min and a slow, linear increase in pressure. Third, postural testing identifies posture-dependent CSF circulation disorders by monitoring ICP changes from the supine to the sitting position. In summary, after excluding acute intracranial hypertension, a pattern of normal or mildly elevated baseline pressure, Rout > 13, persistently elevated nocturnal RAP > 0.6, and prominent B waves suggests shunt-responsive NPH; in contrast, normal or reduced baseline pressure, Rout < 10, persistently low RAP < 0.6, and absence of B waves are more consistent with cerebral atrophy. In addition, increased resistance to CSF outflow has been reported to be associated with benefit from shunt surgery (29). Therefore, as a key functional assessment tool in the differential diagnostic workflow of ventriculomegaly, ICP monitoring can effectively guide individualized treatment decisions. However, invasive procedures and continuous data acquisition pose limitations to clinical practice. Monitoring results may vary depending on surgical techniques and patient characteristics, ultimately leading to inconsistent findings across studies. This highlights the need for multi-center standardization of measurement techniques and the integration of multiple ICP metrics to optimize predictive accuracy. Future directions include developing non-invasive CSF flow assessment and validating combined dynamic metrics across different patient populations.

### 3.3 Cerebrospinal fluid biomarkers

CSF biomarkers represent an important approach for distinguishing hydrocephalus from ventriculomegaly associated with other neurodegenerative disorders. This is particularly relevant in patients with idiopathic normal pressure hydrocephalus (iNPH), because similar ventricular enlargement and clinical symptoms may also be observed in other neurodegenerative diseases, such as AD, VD, Parkinson’s disease, and atypical parkinsonian syndromes—and may even occur simultaneously (30–32). In a retrospective study conducted by Jeppsson et al. (33), CSF concentrations of tau and amyloid precursor protein-derived proteins (sAPPα, sAPPβ, Aβ38, Aβ40, and Aβ42) were found to be lower in patients with iNPH than in healthy controls and patients with non-iNPH disorders, including VD, PD, multiple system atrophy, progressive supranuclear palsy, corticobasal degeneration, AD, and temporal lobe degeneration, whereas monocyte chemoattractant protein-1 (MCP-1) was elevated. A diagnostic model incorporating T-tau, Aβ40, and MCP-1 yielded an area under the curve (AUC) of 0.86 for distinguishing iNPH from other disorders. Another study showed that, compared with healthy controls, CSF T-tau levels were significantly increased in both iNPH and AD, whereas Aβ42 levels were reduced in both conditions. In contrast, CSF P-tau levels were significantly elevated only in AD, but not in iNPH. For differentiating iNPH from AD, a P-tau cutoff value of 47.4 pg./mL showed both high sensitivity and specificity, at 88.7 and 86.7%, respectively (34). More recently, a meta-analysis demonstrated that CSF T-tau and P-tau concentrations in patients with iNPH were significantly lower than those in both AD patients and healthy controls. In addition, Aβ42 levels in iNPH were significantly lower than those in controls, but slightly higher than those in AD. Compared with AD, the overall sensitivity of Aβ42 for distinguishing iNPH was 0.813 (95% CI, 0.636–0.928), whereas that of T-tau was 0.828 (95% CI, 0.732–0.900) and that of P-tau was 0.943 (95% CI, 0.871–0.981) (35). Therefore, given the inconsistent findings across different studies, large-scale, well-designed prospective studies with standardized protocols are still needed for validation.

In addition, several other molecules related to neurodegenerative diseases, neuroinflammation, and demyelination have also been used for the differential diagnosis of iNPH. Neurofilament light chain (NFL) is elevated in many neurological disorders, and multiple studies have reported that compared to healthy controls, iNPH patients have higher concentrations of NFL in their CSF (36). However, Jeppsson et al. (37) reported no difference in NFL concentrations between iNPH patients and healthy individuals. Glial fibrillary acidic protein (GFAP) is a biomarker of astrocyte activity. Studies have reported that the concentration of GFAP in the CSF of NPH patients is higher than that of neurodegenerative dementia patients and healthy individuals (38). However, recent studies have compared the concentration of GFAP in the CSF of NPH patients with subcortical atherosclerotic encephalopathy and found no significant difference (39). Inflammation molecules, including interleukins (IL-1 β, IL-6, IL-10), have been reported to be elevated in iNPH patients and may be involved in the pathogenesis of the disease (40). Lipocalin-type prostaglandin D synthase (PGDS) is mainly involved in prostaglandin metabolism and retinol transfer. Studies have found that its concentration in the CSF of NPH patients is significantly lower than that of AD, depression patients, and healthy individuals (41). In addition, metabolites can also serve as biomarkers for CSF and be detected in the CSF. Nagata et al. (42) found that compared with the CSF of AD patients, the concentrations of serine and 2-hydroxybutyrate in the cerebrospinal fluid of iNPH patients were significantly increased, while the concentrations of glycerol and N-acetylglucosamine were significantly decreased. However, despite a large number of biomarkers being proposed, differences between studies, lack of standardized detection methods, and limited prospective validation have limited clinical applications.

In recent years, peripheral blood biomarkers have also been explored for distinguishing hydrocephalus from neurodegenerative disorders associated with ventriculomegaly or overlapping symptoms. A retrospective study suggested that the neutrophil-to-lymphocyte ratio (NLR) may serve as a promising peripheral biomarker for differentiating neurodegenerative disorders from idiopathic normal pressure hydrocephalus (iNPH) in patients presenting with gait disturbance and ventriculomegaly. NLR was significantly higher in patients with neurodegenerative disorders than in those with iNPH. At a cutoff value of 2.0, NLR differentiated neurodegenerative disorders from iNPH with an area under the curve (AUC) of 0.79, a specificity of 70%, and a sensitivity of 80% (43). Similarly, due to limited research, it is still necessary to design high-quality clinical studies to validate. In the Future, research should establish standardized multi-analyte panels and integrate biomarker data with imaging and CSF dynamics to refine patient stratification.

### 3.4 Clinical symptoms

Clinical manifestations remain a core component of the differential diagnosis between non-hydrocephalic ventriculomegaly and hydrocephalus. However, clinical interpretation becomes particularly challenging in cases of secondary non-hydrocephalic ventriculomegaly and secondary hydrocephalus, such as in patients who develop ventriculomegaly after traumatic brain injury or stroke. In such cases, the symptoms may arise either from brain parenchymal damage caused by the primary disease or from secondary hydrocephalus. Distinguishing between these mechanisms is crucial, because only the latter may benefit from CSF shunting. On the other hand, symptom overlap and similar ventricular enlargement may also occur between iNPH and certain neurodegenerative disorders. Therefore, symptom analysis must take into account the overall pattern, temporal evolution, and treatment responsiveness, rather than relying on isolated manifestations.

Gait disturbance is usually the first clinical symptom of iNPH. The characteristic “magnetic gait” is defined by slowed walking speed, shortened stride length, reduced step height, and impaired dynamic balance, particularly during turning. Importantly, however, gait in patients with iNPH is typically symmetrical. Symptoms may improve after the CSF tap test or prolonged external CSF drainage (44). In contrast, gait disturbance in patients after TBI or stroke is usually multifactorial and may reflect hemiparesis, cerebellar dysfunction, spasticity, or ataxia. These gait abnormalities are often asymmetrical and, unless hydrocephalus is also present, do not show sustained improvement after the CSF tap test or prolonged external CSF drainage. Among neurodegenerative disorders, Parkinson’s disease is characterized by bradykinesia, rigidity, resting tremor, and postural instability (45). Vascular parkinsonism may resemble iNPH, but it is usually associated with extensive ischemic white matter changes (46). Patients with Alzheimer’s disease (AD) typically do not present with gait disturbance in the early stage.

Cognitive impairment in NPH mainly affects working memory, learning, attention, executive function, and processing speed. Behavioral changes are usually mild. Frontal lobe functions, such as executive function, are disrupted because of injury to frontosubcortical projections or subcortical structures and are more severely affected than in AD, whereas memory is relatively less impaired than in AD, as reflected by delayed recall and delayed recognition (11, 47). In contrast, AD is characterized by prominent episodic memory impairment and progressive cortical dysfunction (48). Post-traumatic encephalopathy usually manifests as executive dysfunction accompanied by emotional dysregulation, irritability, depression, or impulsivity (49). Post-stroke cognitive impairment may include focal deficits depending on lesion location (50). One key distinguishing feature is that cognitive impairment in iNPH may improve after different forms of CSF removal, whereas cognitive impairment caused by structural brain injury or neurodegeneration usually remains unchanged.

Urinary incontinence in NPH may be related to right frontal hypoperfusion. Furthermore, urodynamic studies have demonstrated detrusor overactivity, reflecting loss of inhibitory control over the micturition reflex and suggesting underlying autonomic dysfunction. This may further worsen urinary symptoms in patients with NPH. Reduced dopaminergic D2 receptor density has also been reported in NPH and may represent another contributing mechanism of urinary incontinence (51, 52). However, patients with secondary non-hydrocephalic ventriculomegaly may also develop neurogenic bladder and urinary incontinence because of brainstem or spinal cord involvement caused by the primary disease (53). Preexisting urological disorders, such as prostatic enlargement, may further confound clinical interpretation. In general, isolated urinary dysfunction without gait disturbance or cognitive decline rarely indicates hydrocephalus. Improvement of urinary incontinence after the CSF tap test or prolonged external drainage may serve as a useful clue in the differential diagnosis.

Therefore, when clinical symptoms are used to differentiate non-hydrocephalic ventriculomegaly from hydrocephalus, they must be interpreted in combination with the CSF tap test or prolonged external CSF drainage. Whether symptoms improve after CSF removal can not only help clarify the origin of the symptoms, but also provide an important basis for treatment planning.

### 3.5 Differential diagnosis framework

**Figure 1:**
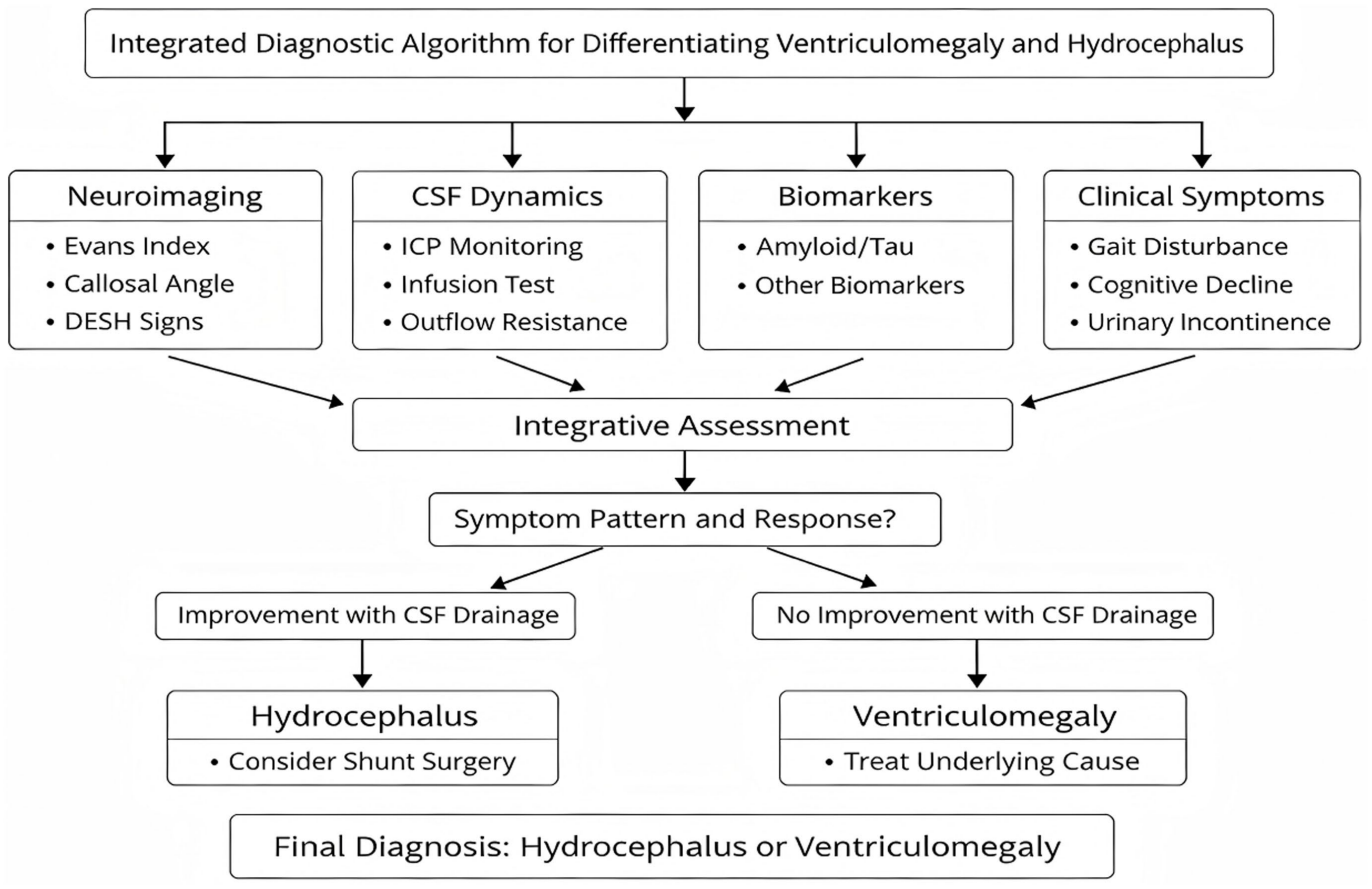
A framework for the differential diagnosis of non-hydrocephalus ventricular dilation and hydrocephalus.

Given the existing clinical challenges in differentiating non-hydrocephalic ventriculomegaly from hydrocephalus, we propose a stepwise and hierarchical diagnostic framework for the evaluation of patients with ventricular enlargement (Figure 1). During the differential diagnostic process, patients may undergo neuroimaging examination, cerebrospinal fluid (CSF) dynamic assessment, biomarker analysis, and clinical symptom evaluation. The results of these assessments are then integrated, and patients suspected of having hydrocephalus subsequently undergo a CSF drainage test. Based on whether symptoms improve after drainage, together with the findings from the aforementioned evaluations, the differential diagnosis between hydrocephalus and ventriculomegaly can ultimately be established.

## 4 Conclusion

In summary, differentiating non-hydrocephalic ventriculomegaly from hydrocephalus requires a comprehensive, multidimensional approach integrating information from multiple diagnostic domains. Structural neuroimaging provides basic information on ventricular morphology, including the EI, CA, and DESH, whereas advanced techniques such as phase-contrast MRI and DTI offer additional insights into cerebrospinal fluid (CSF) dynamics and white matter integrity. These findings help distinguish hydrocephalus from other causes of ventriculomegaly (5, 8, 11, 13–15, 22, 54). Assessment of CSF dynamics through intracranial pressure monitoring and lumbar infusion testing further clarifies whether ventricular enlargement is associated with impaired CSF flow and reduced brain compliance, thereby supporting the diagnosis from a pathophysiological perspective (27–29). Biomarkers, including CSF amyloid-*β* and tau proteins, can help identify neurodegenerative disorders that mimic hydrocephalus. Limited studies have also been conducted on the concentration differences of other cerebrospinal fluid markers (such as NFL, GAFP, PGDS) and many other cerebrospinal fluid molecules, but the results are still contradictory, while peripheral blood biomarkers may provide additional diagnostic value (33–42). Clinical symptom assessment, particularly of gait, cognition, and urinary function, remains essential because it provides functional evidence of CSF-related impairment. When combined with the CSF tap test or prolonged external drainage, it can further support the diagnosis and guide clinical management (44). A critical synthesis of existing evidence highlights the strengths, limitations, and gaps across all diagnostic modalities. For differential diagnosis, neuroimaging and CSF dynamics assessments provide key insights from anatomical and physiological perspectives, while biomarker detection and clinical symptom analysis offer indispensable complementary information from molecular and clinical dimensions.

Current limitations include discrepancies in measurement thresholds, a lack of standardization, and insufficient prospective validation. Future research priorities encompass the multicenter validation of composite imaging and CSF metrics, the standardization of biomarker assays, the development of quantitative symptom assessment tools, and the integration of multimodal data into predictive AI models. These advances may improve patient stratification, reduce misdiagnosis, and ultimately support more personalized management of patients with non-hydrocephalic ventriculomegaly and hydrocephalus.
